# Improving emergency preparedness of subnational vaccine stores in Ukraine: co-creation and implementation of on-site functional simulation exercises

**DOI:** 10.3389/fpubh.2026.1751398

**Published:** 2026-02-16

**Authors:** Stanislav Gaievskyi, Oleg Khomenko, Oleksandra Karkishchenko, Yevgenii Grechukha, Dmytro Nestor, Igor Starychenko, Oleg Benes, Maricel de Quiroz-Castro, Souleymane Kone, Jarno Habicht

**Affiliations:** 1 World Health Organisation Country Office in Ukraine, Kyiv, Ukraine; 2 Center for Research and Training in Disaster Medicine, Humanitarian Aid and Global Health, University of Eastern Piedmont, Novara, Italy; 3 Safe, Affordable, and Effective Medicines for Ukrainians (SAFEMed) Project, Management Science for Health, Kyiv, Ukraine; 4 Ukrainian Public Health Center of the Ministry of Health of Ukraine, Kyiv, Ukraine; 5 World Health Organization Regional Office for Europe, Copenhagen, Denmark; 6 World Health Organization Headquarters, Geneva, Switzerland

**Keywords:** cold chain, conflict, immunization, simulation exercise, Ukraine, vaccine

## Abstract

**Introduction:**

The ongoing war in Ukraine has severely disrupted the health system, with repeated attacks on energy and health infrastructure, as well as damage to transport routes, posing a major threat to maintaining the vaccine cold chain. In this context, the authorities, along with WHO, took action to strengthen the preparedness of subnational vaccine stores to effectively respond to emergencies and minimize the risk of temperature excursions during vaccine storage and transportation.

**Context:**

Ukraine’s vaccine cold chain operates through 25 newly established subnational vaccine stores. These facilities play a critical role in storing and distributing vaccines to lower levels of the health system. Following recent modernization, most stores use WHO-prequalified cold chain equipment, remote temperature monitoring, power backup systems, and specialized vehicles. However, the ongoing conflict exposes them to frequent disruptions in power, logistics, and communications.

**Approach:**

To address this need, we co-created on-site functional simulation exercises. The 7-h programme combined a locally led risk analysis with three progressively complex simulated emergencies, testing responses to equipment failure, prolonged power outages, and transport incidents.

**Implementation experience:**

Between July and November 2025, simulation exercises were conducted in 18 oblasts, revealing recurring gaps in alert systems, backup storage and transport capacity, procedures implementation, and staff training. After the exercises, each store developed a tailored action plan to address these. Participant feedback indicated improved technical readiness, clearer roles and responsibilities.

**Lessons learned:**

Co-created, on-site simulation exercises proved effective and cost-efficient for enhancing cold chain preparedness, offering a replicable model for other public health functions in conflict settings.

## 1 Introduction

Russia’s invasion of Ukraine since 24 February 2022 has profoundly affected all aspects of society, including the health system. The health system has been directly targeted, with over 2,702 attacks reported and 224 health workers killed (1). The indirect effects of Russia’s targeted strikes on critical infrastructure, particularly the power grid, jeopardize vaccine cold chain integrity and significantly disrupt health service delivery (2).

Despite immense pressures, Ukraine’s health system has demonstrated remarkable resilience (3), not only by withstanding multiple shocks but also by advancing key health reforms to sustain and improve service delivery under wartime conditions. By the end of 2024, coverage with the first dose of measles-containing vaccine had surpassed the pre-invasion levels, reaching 91% (4). The polio outbreak that began before the full-scale invasion was successfully closed in 2023 (5). The Health System Development Strategy 2030 was adopted, reaffirming the Government’s commitment to further strengthen the National Immunization Programme (NIP) (6). Immunization coverage indicators are now used as part of performance-based incentives for primary care providers within the Programme of Medical Guarantees (7).

To further consolidate public health functions, the Ministry of Health of Ukraine (MoH) established a network of oblast Centers for Disease Control and Prevention (oCDCs) (8). By 2023, the oCDCs had fully assumed responsibility for NIP management, including vaccine stock management and logistics at the oblast level. Additionally, leveraging resources mobilized during the COVID-19 pandemic, Ukraine upgraded most of its cold chain equipment to WHO-prequalified items (8). These reforms, combined with enhanced infrastructure, have contributed substantially to the demonstrated resilience of immunization services. As further evidence, Ukraine is preparing to expand its NIP to include the HPV vaccine starting in 2026 (9).

The newly established oCDC subnational vaccine stores play a critical role in storing and distributing large volumes of vaccines. Breaches in storage conditions at this level can have serious consequences for the NIP, including vaccine spoilage, supply disruptions, and loss of public trust. The ongoing conflict has further increased the risk of such breaches. In response, the MoH requested WHO support to strengthen the preparedness of these vaccine stores to respond effectively to emergencies and minimize the risk of significant temperature excursions.

Simulation exercises have been recognized as an effective tool for strengthening emergency preparedness and response procedures (10–13). Based on this evidence and considering the practical nature of the challenge, we determined that functional simulation exercises would be the most appropriate approach to address the Ministry’s request. The primary aim of the exercises was to provide targeted training to store staff, and the secondary aims were to assess store preparedness and identify gaps. This paper describes the approach taken to co-create and implement these tailored functional simulation exercises. We believe that our experience may be useful for practitioners seeking to design similar exercises for vaccine stores or other critical public health functions in conflict settings.

## 2 Co-creation of the simulation exercises

To better understand the operational challenges and risks faced by subnational vaccine stores, we first conducted semi-structured interviews with representatives from four subnational stores. These discussions explored past cold chain incidents, anticipated hazards and vulnerabilities, and assessed each region’s overall emergency response capacity, including how national standard operating procedures (SOPs) were adapted locally. We then reviewed national regulations and relevant literature on emergency preparedness and vaccine cold chain management to complement and contextualize the findings (14, 15).

**Figure 1:**
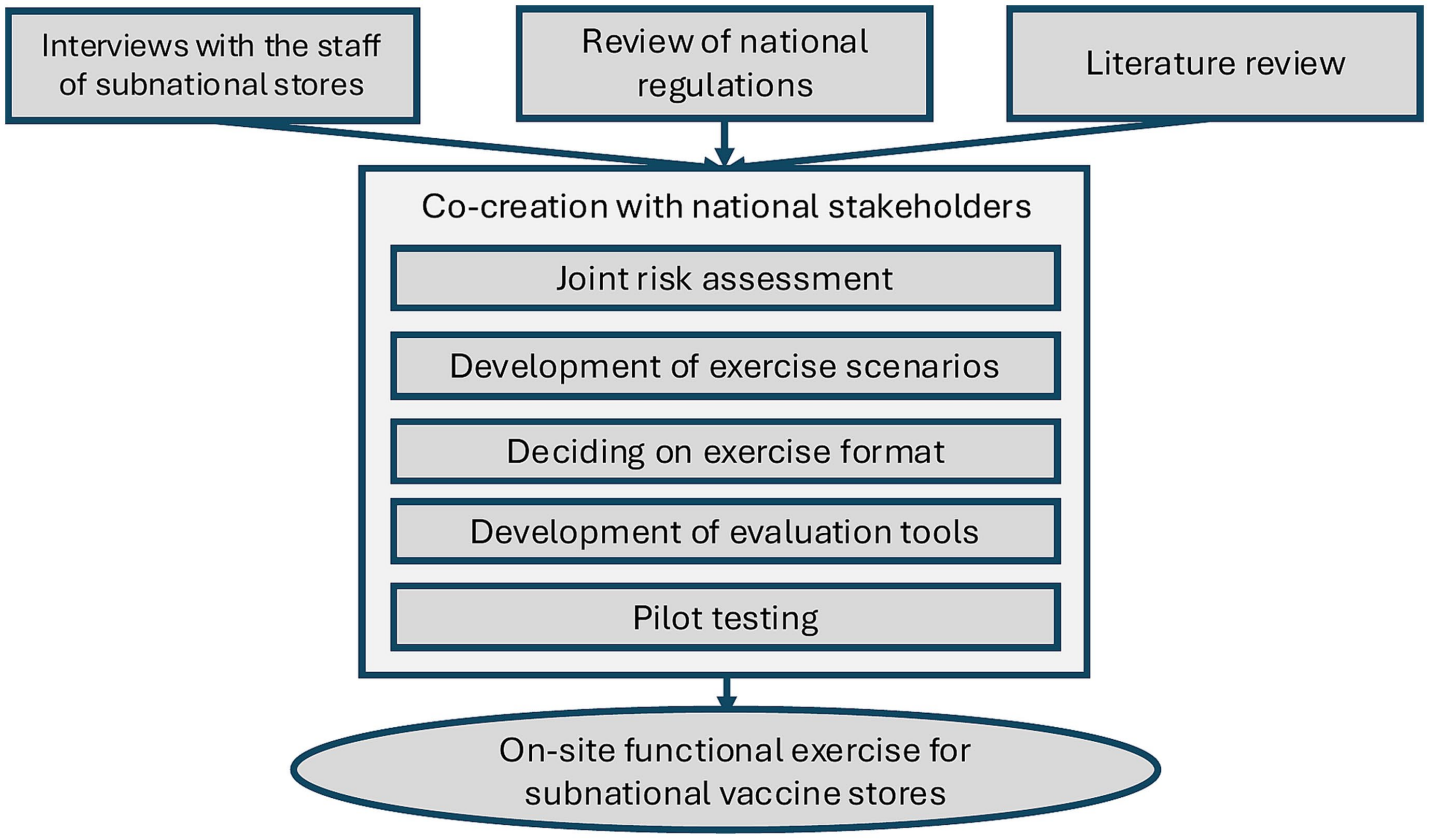
Exercise co-creation process.

Building on these insights, a co-creation process was undertaken jointly by the Ukrainian Public Health Center (UPHC), WHO, and the US-funded pharmaceutical governance project (SAFEMed) to design a practical and context-appropriate functional simulation. The overall co-creation process is illustrated in Figure 1. Through this collaboration, a joint risk assessment was carried out to identify critical vulnerabilities that could compromise vaccine storage conditions at the oblast level.

The results of this assessment guided the co-development of exercise scenarios for subnational vaccine stores, ensuring realism and operational relevance. Using WHO guidance, the team designed the exercise format, prepared supporting materials and participant feedback form (14, 15). The exercise evaluation checklist was developed based on the authors’ experience and was heavily informed by the WHO’s Effective Vaccine Management Guidelines (15). Initial pilot testing enabled refinement of checklists and scenario injects, improving clarity, feasibility, and overall effectiveness before full-scale implementation. In addition to the initial piloting, both the checklist and the scenario injects were iteratively refined by the facilitators after each exercise.

## 3 Exercise structure

The co-creation process resulted in the development of a seven-hour, simulation-based programme consisting of two main components: (1) a locally led risk analysis and (2) three functional emergency response simulations of increasing complexity. Each exercise was facilitated by two WHO staff, with participation from the UPHC representative and partners where possible.

The risk analysis, conducted by store staff with WHO facilitation, enabled participants to identify specific hazards, assess vulnerabilities, and determine the preparedness and response actions needed to mitigate risks. The simulated scenarios included (1) a minor equipment malfunction complicated by curfew restrictions, (2) a prolonged power outage requiring full evacuation of vaccines to alternative storage, and (3) a motor vehicle collision involving a refrigerated vaccine truck. During the exercises, participants performed practical tasks such as calculating available and required cold storage capacity, activating contingency measures, and troubleshooting equipment failures. To ensure realism, local information on SOPs, staffing, equipment specifications, and storage capacities was collected in advance and used to tailor the scenario injects for each store. Template scenarios and the training agenda are provided in the Supplementary material.

Unlike the traditional approach of convening participants from all regions for centralized training, this on-site format enabled realistic, context-specific scenarios, testing of local SOPs, and engagement of all personnel directly involved in emergency response.

Following each simulation, facilitators conducted structured “hot-wash” debriefs to identify procedural and operational gaps and provided targeted feedback. At the end of each session, participants, oCDC leadership, and facilitators jointly developed oblast-specific action plans to strengthen preparedness and cold chain resilience.

Evaluation was conducted from two perspectives: (1) facilitators completed a predefined 18-item observation checklist focused on key structural and operational elements of the response, which informed formal reports submitted to the respective oCDCs and the MoH; and (2) participants provided anonymous feedback on the effectiveness of the exercises and suggestions for improvement. Both evaluation tools are available in the Supplementary material.

## 4 Exercise implementation results

From July to October 2025, we conducted these simulation exercises at 18 of the 25 subnational vaccine stores, prioritizing sites based on their exposure to potential hazards (e.g., power outages) and taking into consideration their accessibility given security constraints. Each exercise involved an average of 12 participants. Core participants included the store manager and the head of the immunization department, but as scenarios evolved, a broader range of staff involved in cold chain incident response were engaged, from the oCDC’s director down to drivers and security personnel. When external institutions, such as providers of alternative storage facilities, were identified in the store’s response plans, they were also engaged to verify their ability to deliver the planned functions and capacities.

**Figure 2:**
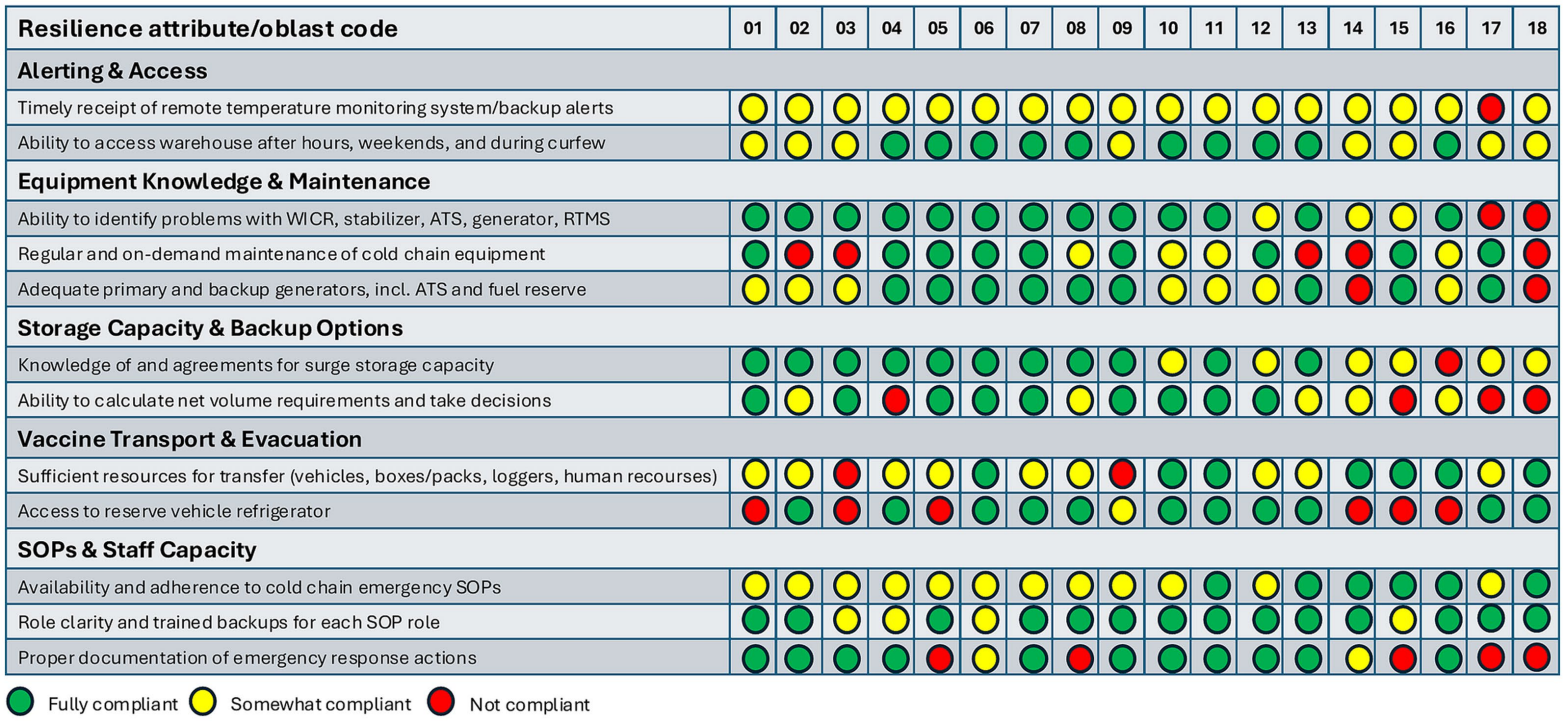
Evaluation of the exercises by oblast.

The 18 simulation exercises conducted across subnational vaccine stores revealed recurring gaps in operational preparedness, captured using the predeveloped checklist and summarized in Figure 2. Key areas of concern included *alert and access*, with delays in remote temperature monitoring system notifications and limited curfew permits hindering timely response; *equipment knowledge and maintenance*, with absence of agreements for regular and emergency equipment servicing; *storage capacity and backup*, including lack of arrangements for alternative storage and insufficient staff skills to calculate storage volumes; *vaccine transport and evacuation*, with insufficient cold boxes, limited backup refrigerated vehicles, and incomplete evacuation planning; and *SOP and staff capacity*, where procedures were not fully aligned with anticipated risks and available routine and surge capacities, and documentation practices were inconsistent. Recommended actions to address these gaps included improving notification systems and curfew access, developing off-site alternative storage arrangements, sourcing additional transport equipment, updating SOPs, identifying surge staff capacities, and providing targeted training. Uptake of the recommendations will be monitored through WHO-facilitated in-person meetings, during which all participating regions will report on progress and exchange experiences regarding barriers and enablers. In parallel, some of the findings have already been taken up by national authorities and WHO in the planning of future cold chain strengthening interventions.

Post-exercise feedback indicated that most participants found the exercise improved their understanding of SOPs, clarified roles and responsibilities, and enhanced skills in calculating storage volumes and using backup equipment. Participants suggested conducting similar exercises for lower-level cold chain facilities, implementing them periodically, and incorporating more complex scenarios while keeping the duration manageable.

## 5 Discussion

Co-created, on-site simulation exercises proved effective and cost-efficient for enhancing cold chain preparedness. Conducting exercises on-site at each sub-national vaccine store enabled tailored, context-specific learning, hands-on testing of technical skills, and targeted feedback from facilitators. The locally led risk analysis improved staff understanding of actual hazards and gaps in response capacities, as well as supported the development of practical, site-specific solutions. Overall, the exercises enhanced familiarity of store staff with SOPs, strengthened coordination with internal and external stakeholders, and helped identify gaps in procedures, equipment, logistics, and training needs. In addition to being effective, this on-site simulation approach proved cost-efficient, as covering facilitators’ travel expenses was considerably less costly than funding travel for large numbers of participants to attend centralized training. The co-creation model used here, involving national authorities, international partners, and frontline staff, provides a template for scaling such exercises across other public health functions.

Regarding the limitations of these exercises, as inherent to the methodology, participants were aware that they were being observed and may therefore have behaved differently than they would in a real emergency. In addition, after the initial exercises, subsequent participating regions were likely aware of the types of emergencies they would face and may have had more time to prepare their responses. It is also important to note that the exercises were designed to assess preparedness and response at the subnational vaccine store level only; therefore, these findings may not fully capture constraints or interdependencies at the national or lower (service delivery) levels of the cold chain, nor broader system-level factors. Nevertheless, despite these limitations, we believe that the exercises proved useful in identifying objective gaps and in providing targeted learning and recommendations to strengthen preparedness, with the potential to contribute to longer-term improvements in cold chain performance.

Our experience highlights the value of conducting functional simulation exercises directly at subnational vaccine stores, demonstrating benefits consistent with broader evidence on simulation-based preparedness (16). Previous research across emergency management, outbreak response, and health logistics has shown that exercises conducted in real operational environments improve the realism of scenarios, enhance team coordination, and more effectively uncover procedural gaps than classroom-based trainings (17). Conducting simulation exercises to improve various health emergency response functions is well documented in the literature and has been applied across diverse contexts (11–13), including in Ukraine (18). However, our experience extends the potential application of this approach to the largely underexplored domain of vaccine stock management and cold chain emergency response in conflict settings.

To our knowledge, this represents the first simulation exercise specifically designed to strengthen vaccine stock management practices. Maintaining the integrity of the vaccine cold chain is essential for sustaining immunization coverage and, more broadly, for ensuring overall health system resilience in conflict settings (19). Our experience in developing and implementing this exercise may offer valuable insights for practitioners seeking to enhance cold chain resilience and other critical public health functions in similar contexts.

## Data Availability

The raw data supporting the conclusions of this article will be made available by the authors, without undue reservation.
