# Supplementary material for: Improving emergency preparedness of subnational vaccine stores in Ukraine: co-creation and implementation of on-site functional simulation exercises
Source: Front Public Health. 2026 Feb 16;14:1751398. doi: 10.3389/fpubh.2026.1751398 (PMC12950732; doi:10.3389/fpubh.2026.1751398)
Supplement: Supplementary file 1 [file Supplementary_file_1.docx]

**Supplementary Material**

**TEMPLATE AGENDA**

| Time | Activity |
| --- | --- |
| 09:00–09:30 | **Welcome and Introduction** |
|  | Participant introductions |
|  | Objectives of the visit and the exercise |
|  | Overview of the agenda |
| 09:30–12:00 | **Risk Analysis of the Vaccine Cold Chain at Oblast Level** |
|  | Using a provided template, participants identified all potential hazards to the vaccine cold chain at the oblast level, including likelihood, potential impact, and necessary actions for prevention, preparedness, and response. |
| 12:00–13:00 | Break |
|  | **Scenarios:** After receiving an injects about the emergency from the facilitators, using provided template participants were asked to outline all necessary response steps. Facilitators assessed the effectiveness and feasibility of the response based on available human and material resources at each oCDC. |
| 13:00–14:30 | **Scenario 1: WICR stabilizer malfunction or WICR cooling systems failure** |
| 14:30–15:30 | **Scenario 2: Power outage** |
| 15:30–16:00 | **Scenario 3: Refrigerated vehicle failure during transport** |
| 16:00–17:00 | **Debrief and Conclusions**  Using template provided, participants documented gaps identified during the simulations and develop action plan to address them. Before closing participants completed online feedback form. |

**FACILITATORS GUIDE**

**Risk Analysis Task Instructions and Template**

This risk analysis table is designed to help your team identify and assess hazards that may affect the integrity of vaccine storage and transport operations at your oblast warehouse. It also supports the development of tailored preparedness and response actions for each identified hazard.

The table can be completed either electronically in Word, with one team member acting as note-taker, or by hand on flipchart paper to encourage group discussion and avoid copying existing materials.

Begin by listing hazards that may compromise cold chain operations at your site. Include hazards already noted in your SOPs, but you are also encouraged to draw from your own experience and knowledge of the local context to identify additional hazards not currently covered in SOPs.

For each hazard, assess both the likelihood of it occurring (e.g., low, medium, or high) and the severity of its impact on vaccine storage or transport if it does occur (also rated as low, medium, or high). Then, provide a brief description explaining the rationale for your assessment. This should include how the hazard might affect the cold chain, any system vulnerabilities, and the available response resources. You should also note any gaps in preparedness or capacity.

Next, define preparedness actions that can be taken in advance of the hazard to reduce its likelihood or impact. Then, describe the response actions that would need to be taken once the hazard has occurred to preserve the integrity of the cold chain.

This exercise is not intended to be perfect but practical. It should reflect the specific hazards and capacities of your warehouse. Facilitators will be available to support you throughout the process. You have 60 minutes to complete the exercise.

| ****Hazard**** | ****Severity / Likelihood**** | ****Description**** | ****Preparedness Actions**** | ****Response Actions**** |
| --- | --- | --- | --- | --- |
| List all hazards that may affect cold chain integrity at your store as well as during transport. List one hazard per row and add as many rows as needed. | Assess the likelihood of the hazard occurring (e.g., low, medium, high) and the severity of its impact if it occurs (e.g., low, medium, high). | Provide a rationale for your assessment of likelihood and impact. Describe the mechanism of impact, vulnerabilities of the system, and available response resources. Note any existing gaps. | List actions that can be taken **before** the hazard occurs to prevent or reduce its impact on cold chain integrity. | List actions that must be taken **after** the hazard has occurred to preserve the integrity of the cold chain. |
| … | … | … | … | … |

**Scenario 1.1. Night-Time RTMS Alert – WICR Stabilizer Malfunction**

**Injects**

1. It’s Friday night at 23:00 when you receive an RTMS alert on your phone, indicating that the temperature inside the walk-in cold room (WICR) has risen to +8.5 °C (if no notification, you check RMTS and recognised that the temperature is above the recommended range). You immediately recognize this as a critical breach in the cold chain and decide to travel to the warehouse to investigate.
2. Upon arrival at the facility, the responding person observes that the WICR is not functioning—there is no sound from the compressor, no interior light, and no visible activity. The building itself has power; lights and sockets are working normally, but the WICR appears to be completely disconnected.
3. During your inspection, you note that the voltage stabilizer, which protects the WICR from power fluctuations, shows evidence of a recent power surge. It seems that in response to a voltage spike, the stabilizer automatically cut off power to the WICR to prevent damage. However, it failed to restore the connection once voltage levels returned to normal, as it is normally programmed to do.

**Task**

Using the template, describe each action taken in response to this incident. Indicate who is responsible for each step, when the action begins, how long it takes, and what resources are used.

**Considerations**

As part of your response, consider who is available to respond at night and whether they are the designated primary cold chain focal point or an alternate. Confirm that the person responding is able to receive RTMS alerts outside regular working hours and is authorized and able to travel during nighttime, including any curfew restrictions that might require prior coordination with local authorities. Specify how the individual reaches the warehouse and whether they have the necessary access to enter and assess the cold room.

You should verify whether the responding person knows how to check and safely restart the stabilizer or organize repairing of broken stabilizer after overloading by voltage and vaccine evacuation, and whether they are trained to assess whether the WICR is safe to re-energize if stabilizer is working well. Clarify how the incident will be documented and explain how any follow-up repairs will be organized, including contact with technical support.

**Scenario 1.2. WICR Cooling Systems Failure**

**Injects**

1. It’s a Tuesday at 16:00 when you receive an RTMS alert indicating that the temperature inside the WICR has risen above +8 °C. Recognizing this as a critical temperature breach, you immediately proceed to the warehouse to investigate.
2. Upon arrival, you find that the main power supply to the facility is fully functional—lights, sockets, and other equipment are operating normally. However, the WICR is clearly not cooling. The RTMS shows a continuing rise in internal temperature, with no signs of stabilization or compressor activity.
3. You inspect both the primary and backup cooling units. Despite power being available, neither is functioning. The cold room is fully stocked with vaccines, and urgent action is needed to prevent a break in the cold chain and loss of valuable stock.

**Task**

Using the template, describe all actions taken to respond to the situation. For each action, specify who is responsible, when it begins, the estimated time required, and what resources are involved.

**Considerations**

As you work through this incident, you are expected to demonstrate your ability to distinguish a refrigeration failure from a power issue, and to troubleshoot both the primary and reserve cooling units. You should identify the location of relevant electrical panels and fuses and contact qualified electrical or refrigeration technicians if needed. Ensure the total vaccine storage volume has been calculated to assess whether on-site alternative capacity exists, and whether it is sufficient. If not, you must determine if a nearby facility has appropriate storage conditions and estimate the time it will take to restore operating temperature either locally or at the alternate site.

If evacuation is required, your response should include a clear transfer plan, including loading and unloading support, cold pack sourcing, and availability of passive cold boxes with adequate capacity. Temperature loggers should be used to monitor vaccine conditions during any transfer. Finally, ensure that the incident is thoroughly documented, and that complete handover records are prepared and retained for accountability.

**Scenario 2. Mass Power Outage**

**Injects**

1. You receive a call from the night security guard reporting a complete power outage in the district and damage to the main generator due to an air attack by drones or missile strike. The cold room and ice-lined refrigerators are undamaged, but the power supply to the cooling system has been cut off. The temperature inside WICR is still stable (+5 °C), but it is only a matter of time before it rises. The cold storage room and ice-lined refrigerators are fully loaded with vaccines.
2. Responsible electrical technician out of communication/ or on leave.
3. Reserve mobile generator placed in another area/or building and didn’t connect.
4. Mobile communications and the internet are experiencing interruptions, most likely due to problems with maintaining stable communication by mobile operator.

**Task**

Using the Annex F template, describe your full response, outlining who is responsible for each action, when it begins, how much time it requires, and what resources are needed.

**Considerations**

As part of your response, consider who is available to respond at night and whether they are the designated primary cold chain focal point or an alternate. Confirm that the person responding can travel during nighttime, including any curfew restrictions that might require prior coordination with local authorities.

After assessment of the causes on site, demonstrate the organisation of contacting outsourced qualified electricians/technicians, if necessary, delivery of a backup generator to the site for quick direct connection to the WICR (cables, connection to a 3-phase generator on a plug-and-play basis)

OR

Assess the decision to evacuate vaccines, calculate the total volume of vaccines and assess the capacity for an alternative location – a backup cold room or a rented semi-trailer refrigerated trailer of appropriate capacity. Organise evacuation, if necessary: transport plan, own or rented refrigerated trucks, , passive cold boxes with ice packs, temperature loggers. Document the incident and keep complete records of the transfer.

**Scenario 3. Refrigerated vehicle failure during transport**

**Injects**

1. You receive a call from a vaccine truck driver reporting a minor vehicle collision while a route to sub oblast office or HCF. The truck remains upright and structurally intact, and the refrigerated compartment is still operational. The temperature inside is stable at +5 °C, and no visible damage to the cargo area is reported.
2. A short time later, during a follow-up call, the driver informs you that the refrigeration unit has stopped functioning. The internal temperature is still holding at +5 °C for now, but it is only a matter of time before it begins to rise. The truck is located in a semi-remote area with no immediate access to technical support or cold chain backup systems. The vehicle is fully loaded with vaccines.

**Task**

Using the Annex F template, describe your full response, outlining who is responsible for each action, when it begins, how much time it requires, and what resources are needed.

**Considerations**

As you plan the evacuation, consider whether a secondary vehicle and driver can be dispatched to the site and whether the total volume of vaccines requiring transfer has been calculated accurately. Assess if your passive cold boxes have sufficient capacity to hold the full load and confirm how and where cold packs will be sourced and conditioned for use. Consider the logistics of loading and unloading support at both the current location and the receiving facility and make sure that temperature loggers are used to ensure continuous monitoring throughout the transfer. Finally, ensure that both the incident and the vaccine handover process are fully documented to maintain traceability and cold chain integrity.

**Response Action Template for Scenarios**

The table can be completed either electronically in Word, with one team member acting as note-taker, or by hand on flipchart paper to encourage group discussion and avoid copying existing materials.

| # | Action Description | Responsible Person/Role | Time Started | Time Required | Resources Required | People Required |
| --- | --- | --- | --- | --- | --- | --- |
| 1 | Describe the specific action being taken to manage the incident. Be clear and concise. Each row should describe a single, concrete step. | Identify the person or role in charge of carrying out this action. This should be based on your actual or expected response structure. | Indicate the actual or expected time that the action begins. Use 24-hour format (e.g., 23:00, 14:15). If unknown, estimate based on the sequence. | Estimate how long the action will take to complete (e.g., “5 mins,” “30 mins,” “2 hours”). Be realistic and consider delays or contextual factors (e.g., night-time travel). | List any tools, equipment, or materials needed to perform this action (e.g., “Phone with RTMS access,” “cold boxes,” “Fuel for generator”). | Specify how many people are needed to carry out this action and any specific skills or roles required (e.g.,“2 people for loading support,” “Driver + assistant”). |
| … | … | … | … | … | … | … |

**EVALUATION CHECKLIST**

| # | Storage resilience attribute | Assessment  (yes/no/partially) | Comment |
| --- | --- | --- | --- |
| 1 | Responsible staff (RS) receives WICR temperature alerts in a timely manner (via RTMS or backup system) |  |  |
| 2 | RS can reach the warehouse during off-hours (incl. curfew arrangements) |  |  |
| 3 | RS can identify and troubleshoot issues with cold chain equipment (WICR, stabilizer, ATS, generator, RTMS, etc.) |  |  |
| 4 | Regular and ad-hoc servicing arrangements for cold chain equipment are in place |  |  |
| 5 | Auto-switch from central power to generator is available and functional |  |  |
| 6 | Primary generator is available and functional |  |  |
| 7 | Secondary generator is available and functional |  |  |
| 8 | Sufficient generator fuel is available at the warehouse |  |  |
| 9 | RS knows net storage capacity of WICR(s), ILR(s), and refrigerated vehicle(s) |  |  |
| 10 | RS can calculate storage volumes by vaccine product/doses/packaging |  |  |
| 11 | Secondary storage arrangements (e.g., backup WICR) exist and are actionable |  |  |
| 12 | Vaccine transfer/evacuation arrangements are actionable, including enough transports, cold boxes, cold packs, manpower. |  |  |
| 13 | Secondary refrigerated vehicle is available and ready in case of primary vehicle malfunction |  |  |
| 14 | Temperature loggers are available in sufficient quantity and used during emergency transport |  |  |
| 15 | Cold chain emergency SOP is available and followed during FX |  |  |
| 16 | RS are aware of their specific roles and responsibilities within SOP |  |  |
| 17 | Each SOP role has a trained secondary/reserve person |  |  |
| 18 | RS can correctly document emergency response actions |  |  |

**FEEDBACK FROM**

Participants were asked to indicate their level of agreement with the following statements on a scale from 1 (fully disagree) to 5 (fully agree):

- The FX helped me understand what to do in case of a cold chain emergency.
- The FX helped me identify necessary modifications to the SOP at my store to make it more effective.
- During the FX, facilitators were able to address all my technical questions and concerns regarding cold chain emergency SOPs.
- The format and structure of the FX were appropriate to achieve its stated objectives.

Participants were also asked to respond to the following short-answer questions:

- List three main topics that you learned during the FX.
- List three topics that you feel were not covered or should have been addressed in more detail.
- Anything else you would like to share? Suggestions?
